# Philosophy in the science classroom: How should biology teachers explain the relationship between science and religion to students?

**DOI:** 10.1007/s11422-020-09997-1

**Published:** 2020-11-20

**Authors:** Peter J. Woodford

**Affiliations:** Centre for Research in Modern European Philosophy, Kingston University, Penrhyn Road, Kingston upon Thames, KT1 2EE UK

**Keywords:** Evolution, Science and religion, Science teaching, Philosophy teaching, Science skepticism

## Abstract

This review explores Thomas Lessl’s “Demarcation as a classroom response to creationism: A critical examination of the National Academy of Science’s *Science, Evolution, and Creationism* (2008).” Lessl’s work examines philosophical debates about the relationship between science and religion from the perspective of communication dynamics between science teachers and audiences skeptical about evolution. His essay raises a number of important points that might help educators craft statements that are less likely to alienate religious students and to entrench any pre-existing opposition to evolutionary science. However, in this review, I raise a number of criticisms of Lessl’s account of the problems with the approach taken by the National Academy of Science. I argue that many of the criticisms of NAS’s approach to demarcation are not well-supported, and even were they to be strong criticisms, they do not justify skepticism toward evolution or science in general. Ultimately, I argue that addressing Lessl’s concerns means creating space for more intellectually rigorous and satisfying discussions of science and religion, but this is not appropriate in a biology classroom that merely wishes to introduce evolution. Addressing these concerns requires making more space for philosophy in the curriculum.

This article is a critical response to Thomas Lessl’s “Demarcation as a classroom response to creationism: A critical examination of the National Academy of Sciences’ *Science, Evolution, and Creationism* (2008).” However, since the issues Lessl raises are both nuanced and important—with respect to philosophy, pedagogy, and the wider cultural dialogue about science and religion—I allow myself the liberty to write at some length beyond the normal genre of a “review” of the article. Lessl’s article focuses on the rhetorical and communicative dynamics of teaching about evolution and religion, and his central argument is that philosophical weaknesses in certain attempts to establish demarcation criteria between science and religion invite religious skeptics of evolution to distrust the good intentions of science educators. His core concern is that educators taking this strategy of demarcation to stave off religious skepticism toward evolution may be further entrenching opposition to science within some religious communities.

Lessl approaches these issues by examining the philosophical assumptions of a pedagogical resource designed by the National Academy of Sciences: *Science, Evolution, and Creationism* (2008) (henceforth, *SEC*). This resource is specifically designed to aid biology teachers in the USA who may encounter religious opposition to evolution in the classroom. Lessl approaches *SEC* as a document firmly entrenched in the “culture wars,” and one that needs to be careful of rhetorically positioning itself (whether intentionally or not) too far on the side of secular naturalism against religious belief. We can distinguish between three separate, but interrelated objectives of *SEC*, as Lessl presents it. The first is to make the case that evolution is—and ought to be regarded as—established scientific knowledge. In other words, it is to defend the claim there is no justifiable skepticism (religious or otherwise) toward the scientific consensus that an evolutionary process of descent with modification, driven by the reproductive advantage of traits, produced the diversity of life on Earth. Second, the authors aim to help students understand the grounds for the epistemic authority of the “textbook” scientific account of evolution. Finally, *SEC* aims to provide educators with intellectual resources to assure students that accepting the textbook account of evolution does not pose a threat to prior religious beliefs, particularly to theistic belief. It is in this third realm where most of the problems lie, but the first two are equally important.

While Lessl’s article briefly touches upon the project of demarcation between science and religion as a tenable philosophical endeavor per se, he is mainly interested in it as a *rhetorical* and *communication strategy*. This focus makes Lessl’s argument more difficult to assess because it rests on claims both about the coherence and defensibility of certain philosophical positions, and about the reasonableness of removing trust and doubting good intent on the basis of various objections. In other words, it is one thing to claim that certain philosophical positions are objectionable; it is another to claim that it is justifiable to doubt the good intentions of those advancing these positions. While it is hard to judge whether Lessl is merely describing how a certain audience is likely to respond, or justifying this response, Lessl’s position seems to be that weaknesses in the demarcation strategy taken in *SEC* do give students grounds to doubt teachers’ intentions. More precisely, they give religious students reasons to suspect that science teachers may covertly be trying to undermine their religious beliefs, perhaps even to impress upon them a version of secular naturalism, as they have perhaps been primed to believe by the religious communities to which they belong.

Lessl’s focus on the rhetorical dynamics of various philosophical understandings of science and religion offers a unique contribution, and a welcome focus toward the cultural contexts in which philosophical arguments are employed and deployed. Given the cultural, and political, tensions between some secular and religious communities in the USA, it is clear that *SEC* is attempting to wade into what can appear to be a minefield. Lessl insightfully shifts our focus toward the effects of various metaphysical and epistemological views on the strained relationship between educational institutions and some religious communities. His attention is mostly on to the context of the USA, and this is important not only because *SEC* was composed by an American scientific institute, but also because the tense relations between “creationist,” biblical literalist religious communities and educational establishments is particularly salient and historically entrenched in the US context. Given the absence of a place for philosophy in the elementary and secondary school curriculum in the USA, there is also less of an opportunity to discuss the relationship between science and religion at a sophisticated philosophical level outside of the biology classroom. This is a point I will come back to later.

Despite the contribution of Lessl’s focus on communicative strategies, I think Lessl is too hard on the demarcation strategy. Many of the objections he raises to *SEC* are irrelevant to the question of why anyone ought to affirm scientific accounts of evolution, and the problems he points out can easily be remedied by small changes in emphasis rather than by reconceiving the overall strategy. Moreover, none of the objections he raises, in my view, justify doubting the good intentions of educators toward religious audiences. Indeed, I think one might interpret the rhetorical motivation behind the demarcation strategy—despite its flaws—precisely as attempting to be as ecumenical and pluralist as possible with regard to divergent metaphysical views (whether atheist, theist, or otherwise). Even if one might object to the precise way this pluralism is articulated in *SEC*, and I agree there is room for improvement here, I read the document as attempting to put metaphysical disputes aside so that students can learn about evolution without the cognitive resistance that they may have been primed to bring with them. This seems to me to be the right intention, and students who affirm suspicions about the motives of educators would be drawing the wrong conclusion.

Of course, Lessl’s point is that *SEC* may be inviting this wrong conclusion by adopting a flawed communication strategy, but even that, I think, is misguided. Lessl focuses almost entirely on the strategy of speakers, rather than on the problematic views and attitudes the audience brings to the classroom. Since educators cannot influence what students bring with them, but only how they respond to students’ prior points of view, it is clear why the focus falls on the strategy of educators. Nonetheless, his analysis shows that an audience composed of religious skeptics of evolution is far from an “ideal” audience. Good intentions need to be found on both sides of the communicative interaction—speaker and audience. An audience can reject the authority or intentions of a speaker for bad reasons, or reasons that are immaterial to the core issue at hand. This is what I think would be taking place if religious skeptics were to reject evolution or distrust the good intentions of science teachers for the reasons that Lessl points out.

There is also a key question that Lessl’s analysis circumscribes, but does not sufficiently address. How, when, and where should sophisticated philosophical discussion of the relationship between science and religion take place in the curriculum? At what point in education should these issues be addressed? Lessl’s presentation of these issues makes clear the interplay between philosophical argumentation, trust, and cultural dynamics around the public perception of science. Nonetheless, his focus on the particular document of *SEC* raises the question of whether it is fair for biology teachers who are introducing evolution to students to shoulder the entire burden of introducing philosophy in order to “win over” uncharitable skeptics—a question to which I will return later. Nevertheless, given that this burden currently falls on the shoulders of biology teachers, there are clearly better and worse ways to approach these issues. Lessl’s critical engagement with *SEC* is constructive; he wants educators to do a better job lest religious students find reasons to resist learning about evolution. Moreover, I infer from the article that Lessl wants *SEC* to do better so that religious students can learn to see that our understanding of evolution might also enrich their religious sensibilities.

The rest of this article supports these points through engagement with the details of Lessl’s criticisms of *SEC*. First, I address Lessl’s discussion of the case for evolution and the epistemic authority of evolutionary science. Second, I turn to Lessl’s points about the compatibility between science and religion. Finally, I discuss some of the more general questions raised by Lessl’s article about how science and religion should be approached in the classroom.

## Philosophy of science and the case for evolution

Lessl positions himself in much of the article as a clever and philosophically astute religious student—one does wonder how many students resemble the one depicted here—and imagines various objections such a student might make to *SEC*. The validity of these potential objections to *SEC*, so Lessl thinks, might lead students to affirm their resistance to instruction on evolution and biology and lose trust in both the expertise and the good intentions of their teachers. Furthermore, and this is the tricky claim, Lessl thinks that students would be justified in such resistance given the current problems with *SEC*. Thus, while Lessl’s unique contribution is to bridge philosophical analysis with an understanding of communicative dynamics, there are clearly “purely” philosophical issues here regarding the strengths and weaknesses of various arguments and their entailments. Lessl identifies three philosophical weaknesses of *SEC* that might give such a student reason to question the expertise and good intention of her teachers: (1) *SEC* is “naively” empiricist, (2) *SEC* equivocates, or contradicts its own claims, (3) *SEC* depicts religion in a patronizing way. The first two weaknesses pertain to the case *SEC* makes for evolution and for the epistemic authority of science, while all three pertain to the compatibility between science and religion. I discuss the authority of science in this section, and turn to science and religion in the next.

The case *SEC* makes for the epistemic authority of science, and for evolutionary science in particular, is basically empirical and empiricist. A broadly Darwinian theory of evolution (broadly here because there remains important debate over details, cf. currents discussions of an “extended synthesis” in Pigliucci and Müller 2010) best explains evidence of the history of life on Earth and patterns of traits and behaviors found across diverse species. I do not wish to rehearse the case for evolution here, and *SEC* does not appear to make this full case since it is merely an introduction to learning about evolution (see Jerry Coyne (2009) for a detailed discussion of the evidence for evolution, even though Coyne is also an outspoken advocate for the view that scientific knowledge of evolution is irreconcilable with religious belief—a view that both Lessl and I reject). What is crucial to Lessl’s case is his claim that *SEC* focuses almost exclusively on the empirical and a posteriori nature of scientific knowledge of evolution, while failing to acknowledge the role of creative thought, theorization, inference, and interpretation. While Lessl’s own epistemology largely remains in the background, one can infer that he endorses something like “critical realism,” in which our a priori concepts and intellectual syntheses of empirically observable states of affairs allow us to grasp a reality “out there.”

Lessl sees an opening for the philosophically astute skeptic of evolution in response to naïve empiricism—but I do not. Lessl argues that *SEC* over-emphasizes the empirical aspects of science so as to make it appear as though scientists just read off the nature of reality by looking at things. Such a view would be “naïve” empiricism, and Lessl thinks that *SEC* is led to adopt this flawed position because it more firmly secures the epistemic authority of science, especially vis-à-vis religion. If scientists are basically just reporting what they “see,” and what anyone can see if they look, then if we look for ourselves we will find the same things. There is just as little room to reject evolution as there is to doubt that there is a table in front of me. While naïve empiricism is a weak position, albeit one that may be prevalent among scientists, especially those without philosophical education, it is certainly overplayed in response to skepticism of evolution. Yet there are two problems with Lessl’s argument here. The first is that the passages he points to in *SEC* as evidence of naïve empiricism are not convincingly interpreted as such. The second is that even if *SEC* is naïvely empiricist, objections to this epistemological theory do not undermine the reasons we have to affirm scientific knowledge of evolution, reasons which are grounded in the empirical evidence that counts in its favor.

One example of such uncharitable reading appears when Lessl examines a somewhat benign statement in SEC: “For more than a century and a half, scientists have been gathering evidence that expands our understanding of both the fact and the processes of biological evolution. They are investigating how evolution has occurred and is continuing to occur” (Quoted in Lessl, 5). Lessl claims that this statement is “empirically tendentious,” meaning that it makes it seem as though our understanding of evolution is “constituted” (a strategically ambiguous term here) wholly by observation (Lessl, 5). However, to read it as such is rather an example of Lessl (or Lessl’s imagined student) squeezing out a “tendentious” and “naively empiricist” reading of *SEC* with brute force.

A further example of this is Lessl’s analysis of the definition of science given in *SEC*: “The use of evidence to construct testable explanations and predictions of natural phenomena, as well as the knowledge generated through this process” (Quoted in Lessl, 7). Lessl writes that “on its face this definition indicates that “explanations,” “predictions,” and “knowledge” are “generated” directly by evidence” (Lessl, 8). To my eyes, this definition does not indicate this at all; the definition is a rather unobjectionable statement. Indeed, the statement uses the terms “construct” and “generate,” which directly indicate the kind of active intellectual contribution, as opposed to mere passive observation, that Lessl insists on. Ultimately, Lessl also affirms the weak basis of this reading:

Naïve empiricism is not overtly advanced in the booklet examined here. Rather, it is an impression that emerges from the inductivist cast given to evolutionary science in its opening chapter and then applied as the authors go on to contrast science and religion and to refute creationism. By persistently foregrounding the evidences that affirm evolutionary explanations, the authors inadvertently slip into language patterns that encourage readers to suppose that evolutionary understanding arises spontaneously from observation (Lessl, 5).

I wonder: what sort of readers would be tempted to suppose this upon hearing the rather basic statements about science in *SEC*?

The more intriguing argument Lessl makes is that problems with naïve empiricism will throw the *motives* that led to this strategy into suspicion. Lessl argues that *SEC* over-emphasizes empiricism and the empirical basis of science *in order* to block certain moves the religious skeptic might make. He writes: “Undoubtedly the NAS authors are not naive empiricists. It is only the persuasive pressures created by religious skepticism that make these patterns expedient … The motive force that perhaps leads to distortion is the fear that the persuasive strength of this demarcation will be weakened if any kind of autonomous power is assigned to the scientific mind” (Lessl, 9–10). This “suspicious” reading of *SEC*’s account of the nature of science is highly questionable. Indeed, a much more plausible interpretation of *SEC*’s emphasis on the empirical character of scientific knowledge is that it is precisely the empirical evidence for evolution that justifies our affirmation of the theory of evolution as true. It is this evidence that gives anyone—especially the skeptic—reason to affirm it as true. In order to defend a suspicious reading of *SEC*’s allegedly naive empiricism, Lessl’s imagined student has to engage in seriously strained, uncharitable readings of the accounts of science given in *SEC*.

It is probable that many skeptics would apply the same brute force to the claims made in *SEC* as are on display in Lessl’s article, but this raises the question of just what kind of attitude the imagined student has: does she have dialectical “good intentions”—i.e. honestly and conscientiously trying to find out how things stand—or is she looking for just any reason to object and doubt the good intention of science teachers? This is important because just as the speaker must respect her audience enough to tell it like it is and make the best case, so too must the audience recognize a good case when it is made. Lessl’s characterization of the audience that produces such readings is telling: “Students who reject evolution on religious grounds will also be prone to doubt the good intentions of science educators, and this distrust will be affirmed by any perceived inconsistency in educators’ claims. Having been forewarned by others who allege that evolutionary science is allied with an anti-religious campaign, these students will also be forearmed with a skepticism that disposes them to treat double meaning as double-dealing” (Lessl, 11). This is probably an accurate characterization of a particular type of audience of *SEC*, one that educators should be aware of, but it also gives us strong reason to doubt that such audiences are well-intentioned toward lessons on evolution. Given the charge of naïve empiricism in *SEC* that Lessl offers on behalf of this audience, one finds one’s doubts here confirmed.

There are a few other issues with Lessl’s depiction of the audience that he is imagining. He writes: “Educators may be right to assume that creationists resist evolution because they fail to appreciate the vital importance of empirical corroboration in science, but inconsistencies are bound to manifest if teachers answer this by assigning powers to observation that observation does not possess. Readers who already doubt evolution will be the first to notice that its theoretical claims are not wholly capable of empirical confirmation or even falsification” (Lessl, 22). There is a serious problem with Lessl’s claim that the evolution is not wholly capable of empirical confirmation or falsification. A further aspect of Lessl’s own philosophical commitments emerges here that show that he may be more sympathetic to skeptics of evolution than he should be. Of course, everything hangs on how we understand the term “wholly confirmed,” but here it appears that Lessl is siding with those who deny that the evidence for evolution is fully convincing. Going into the details of the case for evolution here would require too much digression, even though it is of course in the details of this case that argument is to be won or lost, but can it be that Lessl himself is affirming a theory of confirmation that is “naively empiricist”? In other words, one that holds that we need to directly “see” something occur in order to know that it occurred? It is important for us not to think that science teachers need to answer a form of skepticism so radical that it would require them to demonstrate the existence of the external world before affirming evolution.

The crucial issue, in my view, is that while it is—as a factual matter—important to recognize that scientists do not simply “see” the truth of theories in the way that we “see” that there is a table in front of us, the difference between a “naïve” and, let us say, “critical” account of the nature of science does little to alter the case for evolution. This is because our scientific knowledge of evolution *is*—*and ought to be*—*justified on empirical grounds*. As already mentioned, *SEC*’s insistence on the empirical and evidentiary basis for the adjudication and validation of knowledge claims can also be read an attempt to outline the genuine reasons why anyone ought to believe in evolution. This case for evolution rests on how this theory has born, and continues to bear, itself out against the widest available evidence of the traits and history of life on Earth. Lessl does not address whether *SEC* lists crucial sources of evidence for evolution, or instead if *SEC* only discusses the nature of science—perhaps the rest of the biology course will introduce this evidence and the initial discussion is only meant to “disarm” skeptical students who may not listen. Nevertheless, the case for the contemporary scientific account *should* emphasize the empirical character of science and the empirical basis for evolution. This is a good example of living up to a “discourse ethic,” showing respect for the audience by attempting to articulate the normative reasons why anyone should be convinced of the truth of the claims being made. Students skeptical of evolution and those who affirm it can both benefit from *SEC*’s basic discussion of empiricism and the role of empirical evidence in justifying scientific truth-claims. Such a discussion should not be interpreted as a covert strategy.

In addition to downplaying the role of creative theorization, reasoning, and inference in science, Lessl also charges that *SEC* “equivocates.” Here, his central claim continues to revolve around the question of trust and credibility: “My specific concern here is with how equivocation may affect the perceived credibility or ethos of science educators. Educators who press empirical demarcations too hard will inevitably paint themselves into corners of contradiction, and skeptical readers who take notice will have cause to doubt their good intentions” (Lessl, 3). Again, Lessl’s aim is not really to assess the cogency of objections to various aspects of *SEC*, it is rather to suggest that various philosophical shortcomings of *SEC* give room to students to distrust the good intentions of teachers. Here, the main example of equivocation in *SEC* is the treatment of the term “theory.”

Lessl argues that *SEC* paints itself into a corner in its response to the well-known, but flawed, objection to evolution that it is “just a theory.” In order to counter this objection, *SEC* appears to alter the definition of “theory” so as to make it seem as though the term “theory” when applied to evolution diverges from its ordinary meaning. Lessl is right to point out that this is a misguided strategy that gives far too much credit to the “it’s only a theory” objection. The “it’s only a theory” objection is simply *bad*, and—as Lessl recognizes—equally guilty of failing to understand the relationship between theory and empirical evidence in scientific reasoning. The right way to put things is that empirical evidence gives us conclusive reason to regard the theory of evolution as a *true* theory. Moreover, a straightforward entailment of evolutionary theory being true is that evolution *in fact* occurs, and in the way that we think it does. All *SEC* would need to do to respond to this charge is change wording slightly.

Lessl acknowledges this, writing that “[t]he authors might have answered this objection straightforwardly by explaining that scientific claims express a wide range of probabilities, that some are highly speculative and controversial while others, like evolutionary theory, are not. They instead answer with a demarcation that they cannot consistently uphold” (Lessl, 11). Yet he goes on to justify the student’s doubt of the good intentions of the speaker: “Readers who notice this equivocation may find their doubts affirmed if they also notice that the above passage answers the “only a theory” question by dodging it” (Lessl 13). The problem with Lessl’s account here is the ambiguity of his validation of the student’s suspicion. It may be accurate to suppose that students will find the way that *SEC* re-defines the notion of “theory” unconvincing, but why does this give students reason to doubt evolution or the good intentions of teachers? This response also only makes sense in light of the way that some students may be “armed” at the outset against evolutionary science. Just as in the case of the strained interpretation of naïve empiricism, the key point here is that objections to equivocation in the term “theory” give one no reason to doubt evolutionary science. If a student were clever enough to object to inconsistent definitions of “theory” and “fact,” such a response would indicate that she is not clever enough to notice that this problem in no way undermines the basis of evolutionary science. This is again a minor problem, remedied by re-wording things slightly to assert that we have decisive reason to affirm that the theory of evolution is true, and as a result, that evolution has *in fact* occurred.

Again, Lessl’s aim is in part to paint a picture of a certain type of audience, and he may be descriptively correct that perceptions of naïve empiricism and equivocation will lead some students to distrust teachers. But it is important to highlight that none of the imagined objections Lessl cites actually justify this response. The student’s suspicious reaction only makes sense in light of the fact that she is not intellectually “good-intentioned.” Lessl’s analysis does not appear to take the primary aim of *SEC* to be setting out the grounds of the epistemic normativity of scientific accounts of evolution—the case for why we have reason to affirm it as true. Instead, he takes the primary aim to be one of convincing a particular type of uncharitable audience that already rejects evolution and will look for reasons to affirm this rejection. Nonetheless, as it is clearly to the advantage of educators to preempt any such suspicious reaction, Lessl’s suggestion to re-structure wording as necessary should be heeded.

## The compatibility between science and religion

While I am more sympathetic to Lessl’s worries about the dangers of a flawed approach to the compatibility between science and religion, here too I think he is too hard on *SEC*. Moreover, I think Lessl’s concerns lead in a direction that he does not take—namely, to reflection on where philosophy belongs in the curriculum. Lessl insightfully points out that science teachers place themselves in a vulnerable position when they move from the nature of science to the nature of religion and to the “demarcation” between them. Sorting out the relationship between science and religion is a topic that raises philosophical issues beyond the philosophy of science and understanding of scientific methods. Science teachers may not have the same expertise regarding the histories, practices, and philosophies of religious communities as they do about empirical evidence for evolution and scientific methods. Moreover, affirming the compatibility between science and religion means taking responsibility for assuaging any cognitive dissonance that may have been created by forcefully making the empirical case for evolution.

Lessl’s focus on the issue of trust is helpful, and crucial, here. Failing to make a convincing case that science and religion are compatible after forcefully affirming the truth of evolution is somewhat like leaving religious students stranded in an area where they may not know the way back home. Furthermore, as Lessl points out, “followers of these [religious] traditions are likely to perceive that they are being patronized when they find themselves confronted by scientists who presume to explain the nature of religious beliefs that they have made little effort to understand” (Lessl, 4). Of course, students who affirm doubts about evolution because science teachers do not give satisfying accounts of religion, or of the compatibility between science and religion, would still be drawing the wrong conclusion. Understandings of the nature of religion are irrelevant to the case for affirming evolution. But in this case, I agree with Lessl that is far easier to justify the removal of trust due to being patronized, or even abandoned, than for reasons having to do with teachers having an insufficiently “critical” epistemology. The question here is whether teachers need to be pastoral in relation to these issues, giving students some resources to put things back together, and not simply let the chips fall where they may.

Lessl’s main charge here is that *SEC* does not actually show how scientific knowledge of evolution can be harmonized with religious (theistic) belief. Instead, he charges that it engages in superficial “hand-waiving,” assuring students that many religious believers find no conflict here without actually outlining the rational basis for such assurance. Worse, Lessl charges that *SEC* undermines the rational basis upon which this compatibility might be affirmed. This is clearly at the heart of Lessl’s worry about perceived naïve empiricism in a document that intends to argue for the compatibility between science and religion. A naively empiricist account of science would likely also lead to a crude—but all too familiar—demarcation, according to which science deals with observable and verifiable aspects of the world, while religion deals with unobservable, transcendent, and supernatural aspects. Thus, science deals with confirmable “fact,” while religion deals with non-rational “belief” or “faith.” Jerry Coyne is a good example again here because while he provides a detailed account of the evidence for evolution in one book, in another he typifies this “scientistic” demarcation between science and religion, and goes on to reject their compatibility on the ground that religions give us theories for which there is no evidence (Coyne 2015).

Lessl is justified in his concern that if this is the subtext of a document that affirms the compatibility between science and religion, this affirmation would appear hand-waiving and disingenuous. But the problem here too is that Lessl’s interpretations of statements made in *SEC* are uncharitable, and his (or the imagined student’s) expectations of what such a document can and should accomplish are unrealistic. For example, *SEC* states:

Science and religion are based on different aspects of human experience. In science, explanations must be based on evidence drawn from examining the natural world. Scientifically based observations or experiments that conflict with an explanation eventually must lead to modification or even abandonment of that explanation. Religious faith, in contrast, does not depend only on empirical evidence, is not necessarily modified in the face of conflicting evidence, and typically involves supernatural forces or entities. Because they are not a part of nature, supernatural entities cannot be investigated by science. In this sense, science and religion are separate and address aspects of human understanding in different ways. (Quoted in Lessl, p. 20)

This appears to me to be a fairly unobjectionable, and even constructive, way of acknowledging real differences between science and religion while validating religious (theistic) belief. However, Lessl reads this statement very differently: “[i]n proposing that science deals with evidence and religion does not, the authors invoke the familiar notion that religious convictions have extra-rational bases, and one can certainly think of many ways in which this is true. But, were this uniformly true, it would be impossible to say that religion and science could be compatible—unless one means by “compatible” something more like mutually irrelevant” (Lessl, p. 20). The above quote suggests that religious belief is a conclusion drawn neither from empirical evidence *only*, nor from the results of experiments. It does not reject the possibility of any rational basis for religious belief. Lessl’s proposed reading strikes me as an over-interpretation; it reads into this document a Coyne-like position that is not actually there.

This over-interpretation is also evident as Lessl comes to his strongest conclusions: “The clean science-religion demarcations employed here cannot answer this audience’s most crucial objection, their belief that evolutionary science contradicts the Abrahamic doctrine of creation” (Lessl, p. 22). He goes on to affirm that “[p]ersuasion can only succeed if speaker and audience can find some substantial point of agreement upon which to begin, and in the present instance this must be the agreement, however tentative, that nature as described in evolutionary terms might also be regarded as a divine creation. To ask religionists to assume otherwise is tantamount to asking them to forfeit their faith, and, implicitly at least, this is what *SEC* demands” (Lessl, p. 23). Here we seem to find ourselves at the heart of Lessl’s worries, and of his argument. *SEC* denies that nature, as described in evolutionary terms, might rationally be conceived as a divine creation and, thus, it forces upon students a binary choice between evolutionary science and theistic belief. More strongly, it actually undermines theism as a rational view in the face of evolutionary science.

There are two ways that *SEC* might respond to this objection, both of which are to some extent behind Lessl’s worries. The first is simply to clearly distinguish between creation*ism* and the doctrine of creation. Creation*ism* affirms the literal truth of biblical narrative of creation, while the doctrine of creation takes the biblical account as an allegory for a perhaps ultimately inconceivable “moment” of divine creation. The failure to distinguish these is a serious problem for many discussions of science and religion, particularly those of the “Coyne-like” variety, as it prevents any more sophisticated way of discussing the potentially harmony between them. Lessl wants *SEC* to persuade “naïve” creationists to adopt a more “critical” religious position, while persuading the latter that such a “critical” position is perfectly defensible over-against evolutionary science. Thus, *SEC* must show that science can conflict with certain religious beliefs that enter its domain, but it can neither affirm nor reject religious (again, theistic) belief *tout court*. To my mind, the demarcation strategy is necessary for the case that Lessl wishes to make, and *SEC* appears to make the right case. It shows that some religious beliefs affirm metaphysical truths that are largely inaccessible to the methods of empirical science, and thus not falsifiable by them, while others do not. Nonetheless, here too there are some rhetorical improvements that might be made to better acknowledge the difference between biblical literalism, which does conflict with evolution, and more sophisticated forms of theism, which do not. The point here again is that such improvements would merely change emphasis here and there, but they would not overhaul the demarcation strategy as a whole.

If clearly and emphatically distinguishing between creationism and the doctrine of creation is not sufficient to assuage these concerns, we find ourselves facing a bigger conundrum that Lessl presents us with. In order to satisfy the demand that *SEC* convincingly demonstrate how science and religion can be harmonized in a rational way, it appears that biology teachers would have to present and validate—to some extent—theistic arguments. *SEC* might have to talk about how theists understand the transcendent nature of God and the relationship between creator and creation. Or teachers might have to discuss the tradition of “natural theology,” which is the attempt to reason from features of the natural world to notion of an ultimate, transcendent, divine creator of nature. In terms of a confrontation with evolutionary science, the argument that appears most relevant is the “teleological” argument from design (as opposed to the ontological or cosmological arguments, which also present rational grounds for religious belief). I do not wish to rehearse the details of the design argument, or of contemporary debates around it, but clearly if the argument from design is valid, students have reason to affirm the compatibility between evolution and religion. More strongly, if it is valid, even non-religious students should be brought to affirm the doctrine of creation.

There appears to be another argument that Lessl has in mind that can be inferred from other claims he makes. At one point in his discussion of naïve empiricism, he writes: “Any robust discussion of theory will draw readers’ attention to something profound, that scientific conceptualization enables the mind to know beyond sight’s capacity, but this also comes with the risk of blurring the lines that divide science from other enterprises of inquiry” (Lessl, p. 10). Here, there is the sense that Lessl’s frustration with naïve empiricism is not that it obscures the fact that creative thinking, concept formation, and theorization are crucial for the generation of scientific knowledge, it is that scientific knowledge is itself a “profound” phenomenon in nature. Failing to acknowledge the role of our conceptual, methodological, and intellectual abilities in generating scientific knowledge hides the profound fact that our minds can “see” nature beyond bare observation. This capacity of the mind might be enable it to discover non-empirical, metaphysical, truths, for example. Moreover, the fact that nature can be intellectually grasped by products of evolution may be a natural phenomenon that we do not, as yet, have strong evolutionary or mechanistic explanations for. Perhaps Lessl takes it that religious views of nature are better positioned to explain the existence and possibility of scientific knowledge of nature than the available non-theistic accounts are. For example, this is what Alvin Plantinga’s evolutionary argument against naturalism holds (See Beilby 2002). My aim here is not to defend either of these arguments, but rather to use the design argument and Plantinga’s evolutionary argument against naturalism as examples of reasoned cases for affirming theistic belief in light of evolution.

Lessl does not recognize such arguments directly, but one can infer that his frustration with *SEC* is that its alleged “naïve empiricism” removes crucial legs upon which stronger arguments for the compatibility between science and religion might be made to stand. However, presenting these grounds should indicate three features that complicate Lessl’s analysis. First, it does not seem appropriate to debate the merits of theistic arguments in a biology classroom in which the aim is to introduce evolutionary science. Second, doing so might prove antagonistic, since the implication of presenting rational grounds of theistic belief is that others also be persuaded to accept these grounds. Thus, it might then appear that science teachers are attempting to persuade students to be theists. Finally, biology teachers should not be expected to engage with these issues in the philosophy of religion in the sophisticated way that they demand, certainly not in the context of an introduction to evolution.

## Philosophy in the science classroom

Here we have run into a difficult problem that Lessl’s article helps bring into view. It would be hard to defend the claim that science teachers should discuss the merits of design arguments or Plantinga’s evolutionary argument against naturalism, or even dive into the history of rationalist and empiricist theories of knowledge and their metaphysical assumptions. Against the impossibility of a deeper investigation into the history and philosophy of science and religion, the demarcation strategy in *SEC* can be seen in a different light, one that shows that it rests on good intentions: it allows *SEC* to be as ecumenical as possible with respect to diverse metaphysical views (whether religious or secular) while still insisting on our knowledge of evolution. Indeed, I would argue that this is precisely the motivation and intention that science teachers should have in this context. Of course, one might have a problem with metaphysical pluralism as a tenable philosophical position in its own right, and there are lively discussions of this as well in the philosophy of science and the philosophy of religion, but clearly this too is a topic that should be addressed in a philosophy classroom and not by science teachers simply wishing to “disarm” religious skepticism in order to teach evolution.

There are complex historical and cultural forces that have led to the build-up of tension around the “moment” of introductory lessons on evolution, but in my view Lessl’s article shows this moment cannot satisfactorily resolve all of the issues that it takes on. Issues underlying the relationship between science and religion are complex. Indeed, professional philosophers and theologians work hard to understand these questions in all of their complexity and to offer satisfactory answers to them—and there is reasonable disagreement about the answers. Even though academic philosophers and theologians may stake out and defend strong positions on either side, I regard most debates in this area as open-ended and without clear resolution. These debates have to deal with questions as varied as what counts as evidence; Is there a single scientific method, or are there methods; what limits might there be to scientific methods; how are science and religion different; where does science end and metaphysics begin; and what are the grounds of diverse religious beliefs? Some of these are clearly internal to science and scientific thinking, and thus belong in an introductory lesson on the nature of science. Yet some are peripheral, and while I agree with Lessl that philosophy should be a part of any education, and science education in particular, I am less sure of where, when, and how it should be introduced (see Nagel 2008 for an insightful discussion of these issues). Moreover, there are questions central to any “demonstration” of the compatibility of science and religion that clearly aren’t internal to science, and that require engaging in descriptive and constructive *theology*: what do, and should, adherents of theistic religions hold to be true about God, nature, and human origins based on their own sources of authority? What is the strongest, or best, theistic response to scientific accounts of the evolutionary process?

There is clear, and I think vastly underappreciated, pedagogical value in addressing these questions in a sophisticated way, but the list should show that *adequately* addressing them is a task too big for a short introduction to evolutionary science. Seen in this light, the task that the authors of *Science, Evolution, and Creationism* have set themselves is extremely ambitious. This document in no way satisfactorily exhausts questions about the compatibility between science and religion—nor can it—and it should acknowledge this. This raises further key questions that Lessl does not address: if scientists cannot reasonably be expected to offer fully satisfactory lessons in the history and philosophy of science or philosophy of religion, where and when should students learn about rigorous attempts to think about the epistemological foundations of science and the relationship between science and religion? To me, these are the big questions at the heart of Lessl’s article, and my core take-away from Lessl’s analysis is that if educational institutions want to take on science skepticism (whether religious skepticism of evolution, or even concerning topics like vaccination and climate change) the topic of evolution and religion might serve as an alarm bell that philosophy instruction needs to be taken seriously. It will have to be a core part of the remedy. The lack of space or attention to philosophical training is a problem not only for introductory lessons on evolution, but also throughout more specialized science education at the University level.

## Conclusion

My aim here has been to reflect on Lessl’s critique of the demarcation strategy in *SEC*. I have argued that *SEC* does not appear to be naïvely empiricist, or to reject the possibility that reflection on nature might inspire religious belief. Lessl’s criticisms will help scientists craft statements that better preempt such objections, but this will only require minor changes in wording and emphasis. I have argued that the objections Lessl lists are tangential to the core question of why anyone ought to affirm, and therefore learn about, scientific knowledge of evolution. Thus, they are tangential to the case any good intentioned teacher ought to make against skepticism. As a result, the fact that students might be led to reject evolution or the good intentions of science teachers only makes sense as the response of an uncharitable audience looking for reasons to resist lessons on evolution. Lessl may be right that this is the response such an audience will have, but it is not the response it ought to have.

This leads to a final, and important, point that Lessl makes about science skepticism. He argues that “[s]cholars have been in agreement from classical times down to the present that audience judgments of ethos tend to trump all other considerations” (Lessl, p. 22). He argues that this underlies the communicative dynamics of teaching about evolution, and that this is true from skepticism about evolution to climate change and vaccination. In the current climate of the COVID-19 pandemic, we have all the more reason to think deeply about the nature of science and the sources of science skepticism. However, we should also be deeply troubled by this claim, given Lessl’s descriptions of an audience already primed to reject the good intentions of speakers, an audience that then affirms this prior rejection on the basis of criticisms that do not touch upon the core issue at hand. It is hard to imagine what rhetorical and communicative strategy is appropriate for countering such a response, especially if making the correct case is not sufficient.

An appreciation for the limits of the context in which the strategy of *SEC* is being deployed leads me to think that the demarcation strategy is, actually, a good one. Lessl himself cites evidence that this strategy is effective in disarming religious skepticism toward evolution. This strategy allows for an initial, introductory presentation of real differences between science and religion that can allow educators to put philosophical and religious concerns to the side in order to focus on biology—which should be the aim of such an introductory discussion of science and religion. The discussion in *SEC* is far from exhaustive, but it is good enough to do the job. The demarcation strategy is especially fitting for a limited context in which considering the validity of arguments for or against theism would not be appropriate, and science teachers may not be best suited to lead a discussion on the validity of these arguments.

Lastly, there is also a minor side benefit that might be had if Lessl’s call to improve how we approach science and religion in the science classroom is heeded. A metaphysically humble discussion of science and its limits that opens students to the world of philosophical debate over science and religion may spark student interest in philosophy, and in digging into questions about metaphysics and epistemology in a deeper way. Certainly, I think educational institutions at all levels should open students to sophisticated work in philosophy. Of course, philosophy is often demanding in terms of abstract thinking, logical analysis, and critical reasoning. For this reason, it is understandable that it is usually left for higher education in the USA. Yet all too often, students can leave even University without any philosophy at all. This is problematic given that if a sophisticated discussion of science and religion is to be had, philosophy is the discipline in which the core issues can and need to be faced.
